# Complete genome sequence of *Salinicoccus halodurans* H3B36, isolated from the Qaidam Basin in China

**DOI:** 10.1186/s40793-015-0108-8

**Published:** 2015-12-01

**Authors:** Kai Jiang, Yanfen Xue, Yanhe Ma

**Affiliations:** State Key Laboratory of Microbial Resources and National Engineering Laboratory for Industrial Enzymes, Institute of Microbiology, Chinese Academy of Sciences, Beijing, China; University of Chinese Academy of Sciences, Beijing, China

**Keywords:** Qaidam Basin, Moderately halophilic, *Salinicoccus halodurans* strain, *Staphylococcaceae*, Genome sequencing

## Abstract

*Salinicoccus halodurans* H3B36 is a moderately halophilic bacterium isolated from a sediment sample of Qaidam Basin at 3.2 m vertical depth. Strain H3B36 accumulate *N*^α^-acetyl*-*α*-*lysine as compatible solute against salinity and heat stresses and may have potential applications in industrial biotechnology. In this study, we sequenced the genome of strain H3B36 using single molecule, real-time sequencing technology on a PacBio RS II instrument. The complete genome of strain H3B36 was 2,778,379 bp and contained 2,853 protein-coding genes, 12 rRNA genes, and 61 tRNA genes with 58 tandem repeats, six minisatellite DNA sequences, 11 genome islands, and no CRISPR repeat region. Further analysis of epigenetic modifications revealed the presence of 11,000 m4C-type modified bases, 7,545 m6A-type modified bases, and 89,064 other modified bases. The data on the genome of this strain may provide an insight into the metabolism of *N*^α^-acetyl*-*α*-*lysine.

## Introduction

Moderately halophilic bacteria are a group of halophilic microorganisms that grow optimally in media containing between 3 % and 15 % (w/v) NaCl. These bacteria exhibit strong salt tolerance and are widely distributed in different high-salt habitats, such as hypersaline soils and lakes, solar salterns, and salted foods [1, 2]. To cope with the hyperosmotic conditions, these microorganisms accumulate large quantities of inorganic ions, such as K^+^ and Cl^−^, or a particular group of organic osmolytes [3, 4], such as sugars (trehalose and sucrose), sugar derivatives (glucosylglycerol and mannosylglycerate), polyols (glycerol and arabitol), phosphodiesters (di-*myo*-inositol phosphate), amino acids (proline, α-glutamate, and β-glutamate), and derivatives (betaine and ectoine) [5–8]. In strain H3B36, which was isolated from subsurface saline soil (3.2-m depth) in Qaidam Basin in the Qinghai province, China, we detected a special compound, *N*^α^-acetyl*-*α*-*lysine, that acts as an organic osmolyte and thermolyte (authors’ unpublished observation). The amount of *N*^α^-acetyl*-*α*-*lysine in the cell was increased and could be accumulated to a high level when strain H3B36 was subjected to salt stress or heat stress. Unlike other compatible solutes, *N*^α^-acetyl*-*α*-*lysine has only been found to date in *Salinibacter ruber* to date, and the molecular mechanisms through which this compound is synthesized and stored are unclear [9, 10].

Based on analysis of the 16S rRNA gene sequence, this strain is most closely related to *Salinicoccus halodurans* W24^T^ (= CGMCC 1.6501^T^ = DSM 19336^T^) [11]. The genus *Salinicoccus*, which was first described by Ventosa et al. [12, 13], belongs to the family *Staphylococcaceae*. To date, 16 validly named species of *Salinicoccus* have been identified; however, only six genome sequences are available. All species of the genus *Salinicoccus* are defined as moderately halophilic bacteria. These organisms may have potential applications in various fields, including as additives in the food industry; for production of polymer compounds, enzymes, and stress protectants; and in environmental protection and biodegradation [14–19].

To obtain insights into the metabolic pathway of *N*^α^-acetyl*-*α*-*lysine and explore the genome of the *Salinicoccus* spp, we performed complete genome sequence analysis and annotation of *Salinicoccus halodurans* H3B36.

## Organism information

### Classification and features

**Fig. 1:**
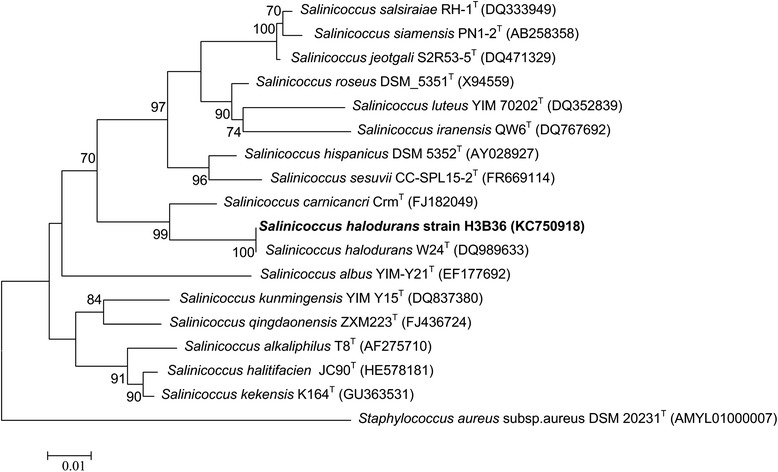
Phylogenetic tree based on the 16S rRNA gene showing the position of *Salinicoccus halodurans* H3B36 relative to other species in the genus *Salinicoccus. Staphylococcus aureus* was used as an outgroup. The analysis involved 18 nucleotide sequences, and there were a total of 1394 positions in the final dataset. GenBank accession numbers for the sequences of each strain are indicated in parentheses. The maximum likelihood algorithm based on the Kimura 2-parameter model was used to construct the phylogenetic consensus tree. All positions containing missing data and gaps were eliminated. Numbers next to the branches represent the bootstrap values obtained by repeating the analysis 1000 times, and values of less than 70 % are not shown at the nodes. The tree is drawn to scale, with branch lengths indicating the number of substitutions per site

Strain H3B36 (Table 1) was isolated from a subsurface saline soil sample (3.2 m depth) from the Qaidam Basin of China by enriching in liquid medium at 37 °C and then plating on agar medium until single colonies were obtained. The 16S rRNA gene sequence of strain H3B36 and other available 16S rRNA gene sequences of closely related species collected from the EzTaxon-e database were used to construct a phylogenetic tree (Fig. 1) [20]. CLUSTAL_X was used to generate alignments [21]. After trimming, the alignments were converted to the MEGA format, and a phylogenetic tree was constructed. The evolutionary history was inferred using the maximum likelihood method based on the Kimura 2-parameter model within MEGA software version 5.10 [22, 23]. Taxonomic analysis showed that strain H3B36 was most closely related to *Salinicoccus halodurans* W24 ^T^ with 99.9 % 16S rRNA gene sequence identity, and as such, strain H3B36 was classified as a strain of *Salinicoccus halodurans*.

**Table 1:** Classification and general features of *Salinicoccus halodurans* H3B36 according to the MIGS recommendations [44]

MIGS ID	Property	Term	Evidence code^a^
	Classification	Domain *Bacteria*	TAS [45]
		Phylum *Firmicutes*	TAS [46]
		Class *Bacilli*	TAS [47, 48]
		Order *Bacillales*	TAS [49, 50]
		Family *Staphylococcaceae*	TAS [48, 51]
		Genus *Salinicoccus*	TAS [12, 13]
		Species *Salinicoccus halodurans*	TAS [11]
		Strain H3B36	IDA
	Gram stain	Positive	TAS [11]
	Cell shape	Cocci	IDA
	Motility	Non-motile	TAS [11]
	Sporulation	Non-sporulating	TAS [11]
	Temperature range	4-42 °C	IDA
	Optimum temperature	28-30 °C	IDA
	pH range; Optimum	5.5-9.0; 7.5	IDA
	Carbon source	Heterotroph	IDA
GS-6	Habitat	subsurface saline soil (3.2 m depth)	IDA
MIGS-6.3	Salinity range;	2-18 % NaCl (w/v)	IDA
MIGS-22	Oxygen requirement	Aerobic	IDA
MIGS-15	Biotic relationship	Free-living	IDA
MIGS-14	Pathogenicity	Unknown	NAS
MIGS-4	Geographic location	China: Qaidam basin	IDA
MIGS-5	Sample collection	2006	IDA
MIGS-4.1	Latitude	37.06 N	IDA
MIGS-4.2	Longitude	94.73 E	IDA
MIGS-4.4	Altitude	2674 m	IDA

^a^Evidence codes**-** *IDA* inferred from direct assay, *TAS* traceable author statement, *NAS* non-traceable author statement (i.e., not directly observed for the living, isolated sample, but based on a generally accepted property for the species, or anecdotal evidence). These evidence codes are from the Gene Ontology project [29]

**Fig. 2:**
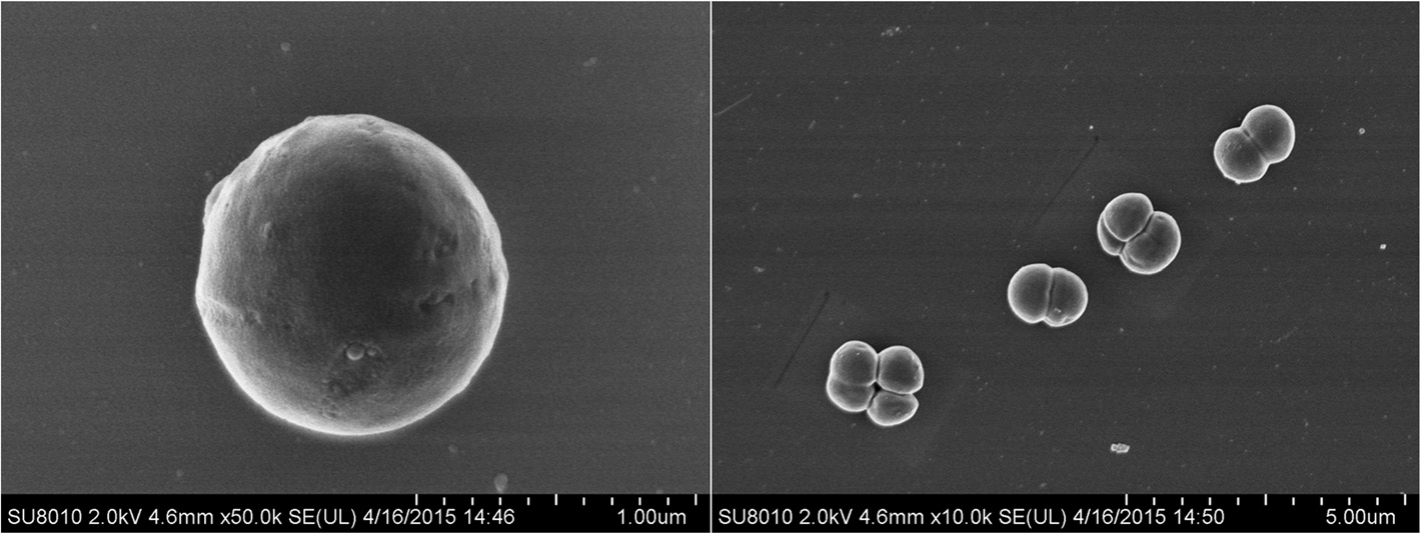
Scanning electron micrographs of *Salinicoccus halodurans* H3B36 using field-emission scanning electron microscopy (Hitachi SU8010, Japan)

The cell morphology of strain H3B36 was determined using scanning electron microscopy (Fig. 2). Microscopically, cells of strain H3B36 were spherical and measured approximately 0.9 μm in diameter. Cells occurred singly or in pairs, tetrads, or irregular clumps at early growth stages. Colonies on GMH agar medium were white, opaque, circular, and slight convex. Cells were able to grow at a temperature range from 4 to 42 °C, with optimum growth observed around 30 °C in GMH medium. Analysis of growth in GMH medium with different NaCl concentrations, the strain grew well when NaCl ranged from 2 to 18 % (w/v) and could not grow in medium without NaCl or with NaCl at concentrations of more than 20 % (w/v). Optimal growth occurred between 4 % and 6 % (w/v) NaCl.

## Genome sequencing information

### Genome project history

*Salinicoccus halodurans* H3B36 was selected for genome sequencing because we observed the presence of a unique compatible solute for protection and potential industrial applications. The complete genome sequence has been deposited in GenBank under the accession number CP011366. Sequencing, annotation, and analysis were performed at WUHAN Institute of Biotechnology, China. The project information and its association with MIGS version 2.0 are shown in Table 2.

**Table 2:** Genome sequencing project information

MIGS ID	Property	Term
MIGS 31	Finishing quality	Finished
MIGS-28	Libraries used	None
MIGS 29	Sequencing platforms	PacBio RS II
MIGS 31.2	Fold coverage	212X
MIGS 30	Assemblers	HGAP2.2.0 workflow
MIGS 32	Gene calling method	Glimmer
	Locus Tag	AAT16
	GenBank ID	CP011366
	GenBank Date of Release	May 11, 2015
	GOLD ID	Gp0114775
	BioProject ID	PRJNA282445
MIGS 13	Source Material Identifier	Strain H3B36
	Project relevance	Environmental and biotechnological

### Growth conditions and genomic DNA preparation

*Salinicoccus halodurans* H3B36 was grown aerobically in GMH medium containing 5 g/L casamino acid, 5 g/L yeast extract, 4 g/L MgSO_4_ · 7H_2_O, 2 g/L KCl, 0.036 g/L FeSO_4_ · 7H_2_O, 0.36 mg/L MnCl_2_ · 7H_2_O, and 60 g/L NaCl, at pH 7.0 (titrated with 1 M NaOH). Genomic DNA from freshly grown cells harvested in the exponential growth phase was extracted using the QIAGEN Genomic DNA Buffer Set and QIAGEN Genomic-tip 100/G according to the manufacturer’s protocols. The prepared DNA was evaluated on a 0.75 % agarose gel to verify the integrity of the molecular weight fragments. Qualification and quantification of the prepared DNA sample was measured with a NanoDrop instrument (Thermo Scientific, Wilmington, MA, USA) and Qubit (Life Technologies, Grand Island, NY, USA) to confirm the suitability of the DNA sample for high-throughput next-generation sequencing.

### Genome sequencing and assembly

The genome of *Salinicoccus halodurans* H3B36 was sequenced using third-generation sequencing technology on a PacBio RS II instrument. The analysis produced a total of 573,153,827 bp, and 54,457 post-filter reads with a mean length of 10,524 bp were obtained. The Hierarchical Genome Assembly Processing pipeline, version 2.2.0, was used to assemble the genome [24–26]. Long reads were selected as the seed sequences for constructing preassemblies, and the other short reads were mapped to the seeds using BLASTR software for alignment, which corrected the errors in the long reads and thus increased the accuracy rating of bases more than 99 %. Based on this analysis, we obtained 95.7 M high-quality reads with an average length of 12,910 bp. Using the overlap-layout-consensus (OLC) algorithms to debug the parameters, we adopted Celera assembler software for assembly. To improve the assembly, the raw data were mapped to the assembled reference sequence to remove any fine-scale errors using Quiver software. Low-depth contigs were then removed, and the rest of the contigs were connected using Minumus2 software. Finally, the data were assembled *de novo* to one final 2,778,378-bp complete contig with 212 × depth of coverage.

### Genome annotation

The RAST Prokaryotic Genome Annotation Server was used to predict protein-coding open reading frames, tRNAs, and structural RNA genes [27]. The Cluster of Orthologous Groups, Gene Ontology, Kyoto Encyclopedia of Genes and Genomes, Swiss-Prot, and Non-Redundant Protein databases were used to annotate the predicted genes [28–32]. Pfam databases were used to predicted genes with conserved domains [33]. Transmembrane helices and signal peptides were identified using TMHMM and SignalP, version4.1, respectively [34, 35]. Tandem Repeat Finder software was used to predict tandem repeat sequences, and Misa software was used to find the minisatellite DNA sequences [36]. Genome islands were analyzed using IslandViewer software, which integrates three software programs (IslandPick, SIGI-HMM, and IslandPath-DIMOB) and combines the Virulence Factor and Antibiotic Resistance Gene databases [37, 38]. In addition, the CRISPR motif was identified using CRISPR II software [39]. Analysis of the raw data was performed to identify loci having epigenetic modifications (i.e., m4C, m6A, and other modification) due to the dynamic characteristics of the raw data [40, 41]. The Restriction Enzyme Database was used to identify the genes involved in the restriction modification system [42].

## Genome properties

**Fig. 3:**
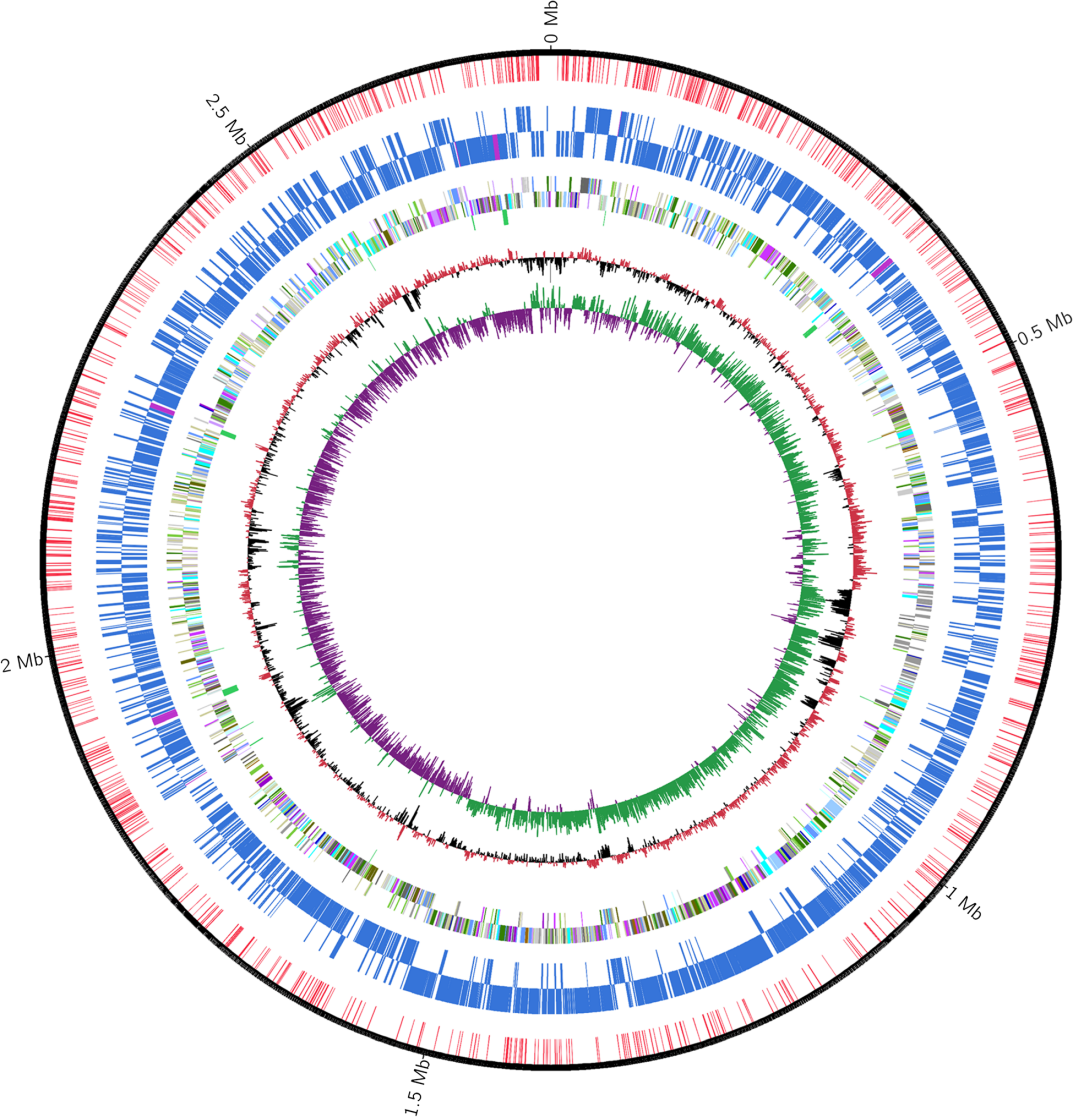
Circular chromosome map of *Salinicoccus halodurans* H3B36. From inner to outer: 1, GC skew (GC Skew is calculated using a sliding window, as (G – C) / (G + C), with the value plotted as the deviation from the average GC skew of the entire sequence); 2, GC content (plotted using a sliding window, as the deviation from the average GC content of the entire sequence); 3, tRNA/rRNA; 4 and 5, CDS (colored according to COG function categories, where 4 is the reverse strand and 5 is the forward strand); 6 and 7, m4C and m6A sites in CDS/rRNA/tRNA (6 is the reverse strand and 7 is the forward strand); and 8, m4C and m6A sites in intergene regions

The complete genome sequence of *Salinicoccus halodurans* H3B36 was found to be 2,778,378 bp and had a G + C content of 44.54 %. No plasmids were found. RAST predicted 2,853 coding sequences, 61 tRNA genes, and 16 structural RNA genes. The predicted CDSs represented 88.79 % of the total genome sequence, with an average length of 864.72 bp. Genome analysis showed that the genome of strain H3B36 contained 58 tandem repeats, six minisatellite DNA sequences, and 11 genome islands. Further analysis of epigenetic modifications revealed 11,000 m4C-type modified bases, 7,545 m6A-type modified bases, and 89,064 other modified bases in the genome. Furthermore, several restriction modification genes were found, with eight belonging to the type I system, three belonging to the type II system, and one belonging to the type IV system. The genome statistics and gene distributions into COG functional categories are presented in Tables 3 and 4, respectively. The circular representation of the bacterial genome was drawn using CGview software (Fig. 3) [43].

**Table 3:** Genome statistics

Attribute	Value	% of Total
Genome size (bp)	2,778,379	100.00
DNA coding (bp)	2,489,753	89.61
DNA G + C (bp)	1,237,616	44.54
DNA scaffolds	1	100.00
Total genes	2,930	100.00
Protein coding genes	2,853	97.37
RNA genes	77	2.63
Pseudo genes	N/D^a^	
Genes in internal clusters	N/D^a^	
Genes with function prediction	2235	76.28
Genes assigned to COGs	2607	88.98
Genes with Pfam domains	2458	83.89
Genes with signal peptides	102	3.48
Genes with transmembrane helices	723	24.68
CRISPR repeats	NA	

^a^ *N/D*, not determined

**Table 4:** Number of genes associated COG functional categories of *Salinicoccus halodurans* H3B36

Code	Value	% age	Description
J	143	5.0	Translation, ribosomal structure and biogenesis
A	0	0	RNA processing and modification
K	206	7.2	Transcription
L	123	4.3	Replication, recombination and repair
B	2	0.1	Chromatin structure and dynamics
D	22	0.8	Cell cycle control, Cell division, chromosome partitioning
V	48	1.7	Defense mechanisms
T	86	3.0	Signal transduction mechanisms
M	129	4.5	Cell wall/membrane biogenesis
N	13	0.5	Cell motility
U	17	0.6	Intracellular trafficking and secretion
O	90	3.2	Posttranslational modification, protein turnover, chaperones
C	174	6.1	Energy production and conversion
G	269	9.4	Carbohydrate transport and metabolism
E	278	9.7	Amino acid transport and metabolism
F	79	2.8	Nucleotide transport and metabolism
H	98	3.4	Coenzyme transport and metabolism
I	139	4.9	Lipid transport and metabolism
P	153	5.7	Inorganic ion transport and metabolism
Q	39	1.4	Secondary metabolites biosynthesis, transport and catabolism
R	277	9.7	General function prediction only
S	222	7.8	Function unknown
-	246	8.6	Not in COGs

The total is based on the total number of protein coding genes in the annotated genome

## Insights from the genome sequence

Genome analysis showed that *Salinicoccus halodurans* H3B36 contained many genes related to the stress response, such as choline and betaine transporters, glycerol uptake facilitator protein, cold-shock protein, chaperones proteins, and others. These genes allowed the strain to cope with different environmental stresses. Experimentation and additional analysis of these genes may help to elucidate the mechanisms mediating the stress response and facilitate the development of *Salinicoccus halodurans* H3B36 for use in industry applications. In addition, several genes encoding hydrolases, including amylase (1), protease (19), pullulanase (2), lipase (3), phosphoesterase (5), and glucosidase (4), were identified in the genome. Hydrolases are highly valuable resources for some specific industrial processes, and hydrolases from various extremophiles may have many advantages [14, 19]. These results indicated that *Salinicoccus halodurans* H3B36 might have the potential for application in industrial biotechnology as a producer of miscellaneous hydrolases.

*N*^α^-acetyl*-*α*-*lysine was found play a key role in protecting *Salinicoccus halodurans* H3B36 cells under different stresses (unpublished observation by Kai Jiang, Yanfen Xue and Yanhe Ma). Genome annotations showed that lysine may be synthesized through the acetyl-dependent diaminopimelic acid pathway in *Salinicoccus halodurans* H3B36. One 8-kb gene cluster containing eight genes was predicted to be involved in *N*^α^-acetyl*-*α*-*lysine biosynthesis. Six genes in the cluster map to enzymes in the acetyl-dependent diaminopimelic acid pathway, including the genes encoding aspartokinase, aspartate-semialdehyde dehydrogenase, dihydrodipicolinate synthase, dihydrodipicolinate reductase, 2,3,4,5-tetrahydropyridine-2,6-dicarboxylate N-acetyltransferase and diaminopimelate decarboxylase. *N*^α^-acetyl*-*α*-*lysine is a derivative of lysine, so this gene cluster may participate in the synthesis of *N*^α^-acetyl*-*α*-*lysine. Further studies are required to verify this assumption and identify the metabolic pathway mediating *N*^α^-acetyl*-*α*-*lysine biosynthesis in *Salinicoccus halodurans* H3B36.

## Conclusions

This is the first report describing the genome sequence of *Salinicoccus halodurans*. The genome size of *Salinicoccus halodurans* H3B36 (2.78 M) is larger than the other sequenced members of genus *Salinicoccus* including *Salinicoccus* sp. SV-16 (2.59 M), *Salinicoccus luteus*DSM 17002^T^ (2.55 M), *Salinicoccus albus*DSM 19776^T^ (2.64 M), *Salinicoccus carnicancri* Crm^T^ (2.67 M), and *Salinicoccus roseus* W12 (2.56 M). *Salinicoccus halodurans* H3B36 has a G + C content (44.5 %) higher than *Salinicoccus albus*DSM 19776^T^ but lower than those of *Salinicoccus carnicancri* Crm^T^, *Salinicoccus* sp. SV-16, *Salinicoccus luteus*DSM 17002^T^, and *Salinicoccus roseus* strain W12 (47.9 %, 48.7 %, 49.1 % and 50.0 %, respectively). Further comparative genomic study shows that the *N*^α^-acetyl*-*α*-*lysine related gene cluster is also found in other sequenced members of genus *Salinicoccus*. The gene cluster in *Salinicoccus* sp. SV-16, *Salinicoccus luteus*DSM 17002^T^, *Salinicoccus carnicancri* Crm^T^, and *Salinicoccus roseus* W12 containing eight genes are similar to that in *Salinicoccus halodurans* H3B36. *Salinicoccus albus*DSM 19776^T^ has a slight discrepancy, which lacks aspartokinase in its gene cluster. The genome of *Salinicoccus halodurans* H3B36 provides important insights into our understanding of the metabolism of *N*^α^-acetyl*-*α*-*lysine. Furthermore, the sequence of *Salinicoccus halodurans* H3B36 provides useful information and may contribute to facilitate applications of genus *Salinicoccus* in industrial biotechnology.

## Abbreviations

HGAP: The Hierarchical Genome Assembly Processing
RAST: Rapid Annotation using Subsystem Technology
